# The operational readiness capacities of the grassroots health system in responses to epidemics: Implications for COVID-19 control in Vietnam

**DOI:** 10.7189/jogh.10.011006

**Published:** 2020-06

**Authors:** Bach Xuan Tran, Men Thi Hoang, Hai Quang Pham, Chi Linh Hoang, Huong Thi Le, Carl A Latkin, Cyrus SH Ho, Roger CM Ho

**Affiliations:** 1 Institute for Preventive Medicine and Public Health, Hanoi Medical University, Hanoi, Vietnam; 2 Bloomberg School of Public Health, Johns Hopkins University, Baltimore, Maryland, USA; 3 Institute for Global Health Innovations, Duy Tan University, Da Nang, Vietnam; 4 Faculty of Medicine, Duy Tan University, Da Nang, Vietnam; 5 Center of Excellence in Behavioral Medicine, Nguyen Tat Thanh University, Ho Chi Minh City, Vietnam; 6 Department of Psychological Medicine, National University Hospital, Singapore, Singapore; 7 Department of Psychological Medicine, Yong Loo Lin School of Medicine, National University of Singapore, Singapore, Singapore; 8 Institute for Health Innovation and Technology (iHealthtech), National University of Singapore, Singapore, Singapore

## Abstract

**Background:**

There is a paucity of data on the operational readiness capacities of the grassroots health system in Vietnam while it plays a vital role as a first-line defense against health emergencies, including the coronavirus disease (COVID-19). This study, therefore, aims to assess the operational readiness capacities of the grassroots health system in response to epidemics and provides implications for controlling COVID-19 in Vietnam.

**Methods:**

An online cross-sectional study using the respondent-driven sampling technique was conducted with 6029 health professionals and medical students in Vietnam from December 2019 to February 2020. The operational readiness capacities of the health system were assessed by the sufficiency of health professionals, administrative and logistics staffs, equipment and facilities, and general capacity of health professionals. Kruskal-Wallis test, Fisher exact test and χ^2^ test were employed to identify the differences among variables. Tobit and censored regression models were operated to determine associated factors.

**Results:**

The operational readiness capacities of the grassroots health system for four assessed criteria were at moderate levels, ranging from 6.3 to 6.8 over 10. In Vietnam, the grassroots health system in rural areas, in the South, and at the district level were more likely to be vulnerable compared to their counterparts.

**Conclusions:**

According to empirical data, this study reveals the vulnerability of the grassroots health system in Vietnam and provides the rationality of prompt and vigorous actions of the Vietnamese Government against COVID-19. Findings also offer useful insights for effective strategies to strengthen the grassroots health system in the long term. In the short term, practicing precautionary measures and mobilizing human resources, as well as medical equipment, are needed to successfully contain COVID-19 in Vietnam.

The coronavirus disease (COVID-19) is a human-to-human infectious disease caused by severe acute respiratory syndrome coronavirus 2 (SARS-COV-2) with initial symptoms similar to seasonal flu such as fever, dry cough, and tiredness [1,2]. It was first detected during an investigation into 44 cases with pneumonia of unknown etiology in Wuhan, China, in December 2019 [3]. Due to its infection and rapid transmission, COVID-19 was characterized as a pandemic by the World Health Organization (WHO) on March 11, 2020 [4]. After four months, on April 18, 2020, it has infected over 2.1 million people with total death reached 146 198 across 213 countries and territories and imposed a great burden on the global health care system and global economy [5-9]. However, public confidence in the health system was associated with less psychological impact during the outbreak and peak of the COVID-19 epidemic in China [10,11]. It is worth noting that developed nations with advanced health systems such as the United States (US) and European countries have been hardest hit due to under-preparedness at the early stage of this pandemic [12-14]. Consequently, these countries are currently suffering from a high number of infection and death cases accompanied by an acute shortage of health professionals, hospital beds and other medical equipment [9,15-17].

To assess the ability of implementation of the International Health Regulations (IHR), WHO required all member states to report their minimum core capacities to detect, evaluate and respond to acute public health risks and events by using the State Party Self-Assessment Annual Reporting Tool (SPAR) [18]. According to IHR SPAR 2018, scores related to operational readiness capacities such as human resources and health service provision of the US, Italy, Spain, and other European countries were substantially higher than those of Vietnam [18]. Nevertheless, Vietnam, a densely populated neighbor of China, has been coping effectively with COVID-19 primarily due to infection prevention, control at the grassroots level, and rapid coordinated responses to this emergent pandemic at the early stage [19]. As of April 18, 2020, the Ministry of Health of Vietnam recorded 268 infected people, of whom 75% were recovered and discharged from the hospital, and no one had lost their life [20]. However, given the underdeveloped health infrastructure alongside with limited financial resources for health care, the Vietnamese Government should continue to pay careful attention to preventive measures, specifically at the grassroots level in the latent stage of the pandemic.

The Vietnamese health care system has a strong focus on disease prevention with the grassroot health networks including 700 district hospitals and health centers, over 8000 commune health stations, and a number of village health collaborators. In each community, there have been involvements of unions of youths and students, women, farmers, and older adults in community health promotion and disaster preparedness. Recognizing the vital role of the grassroots health system in addition to a paucity of data on the capacity of this health system, we conducted this study to assess the operational readiness capacities of the grassroots health system in Vietnam in response to emergent health events such as epidemics including to COVID-19.

## METHODS

### Subjects

The target subjects included health professionals and medical students who were actively involved in national health campaigns by the Vietnam Young Physicians Association. With its establishment in 2009, the association has been pioneering in community health interventions, providing over 2 million free-of-charge health check-ups every year with involvement of over 80 000 physicians and medical students nationwide. The medical training curriculums at Vietnamese medical university requires senior students to implement field practicum and community health education and interventions throughout their programs. Therefore, both health professional and medical students have not only in-depth understanding of the health system, but also experiences at the forefront of management, control, and prevention of any infectious disease outbreaks.

### Study design and data collection

An online cross-sectional study was conducted in 56 cities and provinces of Vietnam from December 2019 to February 2020. The eligibility criteria for this survey were: 1) age from 18 and over; 2) being medical students and health professionals; 3) currently living in Vietnam, and 4) agreement to join in this study by providing the consent online.

First, we sent the survey link to leaders of The Vietnam Young Physicians Association and Youth Unions of several medical and pharmaceutical universities through emails. After that, those leaders sent the link to the seeders of core groups to recruit other participants through the respondent-driven sampling technique. A total of 6029 medical students and health professionals voluntarily agree to participate in this online survey.

### Instruments

We designed a structured questionnaire on SurveyMonkey’s platform. The questionnaire consisted of the socio-demographic characteristic (age, gender, marital status, health system levels, living areas), the occupational characteristics (specialization, health system levels where participants were currently working), we also asked participants that whether or not they had ever participated in epidemic prevention activities at the community level.

To assess the operational readiness capacities of the grassroots health system, we asked participants to assign a score ranging from “not at all” (0) to “completely sufficient” (10) regarding the extent to which four resources in their workplace sufficiently met essential requirements for health care tasks. Those assessed resources consisted of the sufficiency of health professionals, administrative and logistics staff, equipment and facilities, and general capacity of health professionals.

### Statistical analysis

We synthesized and analyzed data by Stata version 12 (StataCorp LLC, College Station TX, USA). Mean, standard deviation (SD) were described for quantitative variables; frequency and percentage were used to describe qualitative variables. Kruskal-Wallis tests, Fisher exact tests, and χ^2^ tests were employed to test the differences between variables. A *P* value *(P)*<0,05 was considered statistically significant. Tobit models and censored regression models were used to determine factors associated with the evaluation of respondents on each component of capacities of the grassroots health system. Forward stepwise selection was used to construct the reduced model that only included independent variables having log-likelihood ratio test *P* < 0.2.

### Ethical consideration

The study protocol was reviewed and approved by the Institutional Review Board of Vietnam Youth Academy. Participation was completely voluntary, and there were no incentives provided. Collected data was saved in a secured system and only served for the study purposes.

## RESULTS

**Table 1** shows that among 6029 respondents, the majority of them were medical students (89.9%), female (71.9%), and single (92.3%). The mean age was 22.9 years old. Most were working or studying at colleges or universities (68.7%), living in the urban (86.1%), and located in the South of Vietnam (64.7%). A larger proportion of respondents (53.9%), most of them were medical students, reported that they had not ever participated in epidemic prevention activities at the community level.

**Table 1:** Socio-economic characteristics of respondents

	Participated in community activities				Total		*P-*value
**Yes**		**No**	
**n**	**%**	**n**	**%**	**n**	**%**
**Total**	2781	46.13	3248	53.87	6029	100	
**Subject:**							
Health professionals	485	17.5	120	3.7	605	10.1	<0.01
Medical students	2282	82.5	3106	96.3	5388	89.9	
**Specialization:**							
Medical specialists	1152	41.9	1119	34.9	2271	38.1	<0.01
General doctors	534	19.4	626	19.5	1160	19.5	
Pharmacists	495	18.0	702	21.9	1197	20.1	
Others	569	20.7	761	23.7	1330	22.3	
**Gender:**							
Male	891	32.0	805	24.8	1696	28.1	<0.01
Female	1890	68.0	2443	75.2	4333	71.9	
**Marital status:**							
Single	2392	86.3	3158	97.5	5550	92.3	<0.01
Living with spouse	335	12.1	47	1.5	382	6.4	
Others	44	1.6	35	1.1	79	1.3	
**Health system levels:**							
Central	338	12.3	295	9.2	633	10.7	<0.01
Provincial	509	18.5	432	13.5	941	15.8	
District	181	6.6	104	3.3	285	4.8	
College/University	1723	62.6	2363	74.0	4086	68.7	
**Living area:**							
Urban	2352	85.1	2803	87.0	5155	86.1	<0.01
Rural	411	14.9	419	13.0	830	13.9	
**Region:**							
Northern	852	31.5	837	26.5	1689	28.8	<0.01
Central	221	8.2	160	5.1	381	6.5	
South	1631	60.3	2159	68.4	3790	64.7	
**Age group:**							
Under 25	2165	82.1	2937	96.5	5102	89.8	<0.01
25 and above	473	17.9	108	3.6	581	10.2	
	**Mean**	**SD**	**Mean**	**SD**	**Mean**	**SD**	***P***
**Age**	22.9	6.0	20.7	2.5	21.7	4.6	<0.01

n – number, SD – standard deviation

**Table 2** reveals the operational readiness capacities of the health system regarding four mentioned criteria. The mean scores of assessed criteria were at a medium level, ranging from 6.3 (the sufficiency of equipment and facilities) to 6.8 (general capacity of health professionals). Significant differences in all criteria were found in two categories, including health system levels and living areas. Within the health system levels category, respondents studying or working at colleges or universities and central-level hospitals reported higher scores than those working at provincial and district levels. Regarding living areas, the capacities of the health system were rated higher by those living in the urban compared to their rural counterparts.

**Table 2:** Capacity of the health system in responses to epidemics (range 0 -10)

	Sufficiency of health professionals			Sufficiency of administrative and logistics staff			Sufficiency equipment and facilities			General capacity of health professionals		
**Mean**	**SD**	***P*-value**	**Mean**	**SD**	***P*-value**	**Mean**	**SD**	***P*-value**	**Mean**	**SD**	***P*-value**
**Total**	6.6	2.2		6.6	2.2		6.3	2.3		6.8	2.2	
**Subject:**												
Health professionals	6.4	2.1	<0.01	6.7	2.4	0.13	5.8	2.2	<0.01	6.3	2.0	<0.01
Medical students	6.7	2.3		6.6	2.2		6.3	2.3		6.9	2.2	
**Specialization:**												
Medical specialist	6.6	2.3	0.85	6.7	2.3	0.53	6.3	2.3	0.05	6.8	2.2	0.25
General doctor	6.6	2.2		6.6	2.3		6.3	2.3		6.9	2.1	
Pharmacist	6.7	2.2		6.6	2.1		6.2	2.2		6.8	2.2	
Others	6.6	2.3		6.7	2.3		6.4	2.3		6.9	2.2	
**Gender:**												
Male	6.6	2.3	0.66	6.6	2.2	0.66	6.3	2.3	0.35	6.8	2.2	0.05
Female	6.6	2.2		6.6	2.2		6.3	2.3		6.9	2.1	
**Marital status:**												
Single	6.6	2.3	0.19	6.6	2.2	0.01	6.3	2.3	<0.01	6.9	2.2	<0.01
Living with spouse	6.5	2.1		6.9	2.3		6.0	2.1		6.4	1.9	
Others	6.3	2.4		6.3	2.5		5.9	2.4		6.6	2.4	
**Health system levels:**												
Central	6.7	2.2	<0.01	6.7	2.2	<0.01	6.3	2.2	<0.01	6.8	2.1	<0.01
Provincial	6.5	2.3		6.7	2.3		6.3	2.3		6.7	2.2	
District	6.1	2.2		6.3	2.6		5.5	2.3		5.9	2.1	
College/University	6.7	2.2		6.7	2.2		6.4	2.2		7.0	2.1	
**Participated in community activities:**												
Yes	6.7	2.2	0.30	6.7	2.2	0.01	6.3	2.2	0.26	6.9	2.1	0.86
No	6.6	2.3		6.6	2.3		6.3	2.3		6.8	2.2	
**Living area:**												
Urban	6.7	2.2	<0.01	6.7	2.2	<0.01	6.4	2.2	<0.01	6.9	2.1	<0.01
Rural	6.2	2.2		6.4	2.3		5.9	2.3		6.4	2.2	
**Region:**												
Northern	6.7	2.2	0.11	6.9	2.2	<0.01	6.4	2.3	<0.01	6.9	2.1	<0.01
Central	6.5	2.1		6.6	2.2		5.9	2.2		6.5	2.1	
South	6.6	2.3		6.6	2.2		6.3	2.3		6.9	2.2	
**Age group (years):**												
Under 25	6.7	2.2	<0.01	6.7	2.2	0.26	6.4	2.2	<0.01	6.9	2.1	<0.01
25 and above	6.4	2.1		6.7	2.4		5.8	2.2		6.3	2.0	

SD – tandard deviation

Factors associated with the capacity of the grassroots health system are indicated in **Table 3**. Living in rural areas (Coefficient (Coef.) = -0.52, 95% confidence interval (CI) = -0.73; -0.31), working at district level hospitals (Coef. = -0.52, 95%CI = -0.89; -0.14), and living in the South (Coef. = -0.22, 95% CI = -0.37; -0.06) were negatively associated with capacity of health system for all assessed criteria.

**Table 3:** Associated factors with the evaluation of capacities of the grassroots health system in responses to epidemics

	Sufficiency of health professionals		Sufficiency of administrative and logistics staff		Sufficiency of equipment and facilities		General capacity of health professionals	
**Coef.**	**95% CI**	**Coef.**	**95% CI**	**Coef.**	**95% CI**	**Coef.**	**95% CI**
**Subjects** (Medical students vs health professionals)							0.36	-0.09; 0.81
**Living area** (Rural vs urban)	-0.52***	-0.73; -0.31	-0.37***	-0.59; -0.16	-0.45***	-0.66; -0.24	-0.50***	-0.70; -0.29
**Marital status** (Living with spouse vs single)	0.39*	-0.03; 0.82	0.43***	0.12; 0.74	0.40*	-0.01; 0.81	0.36*	-0.05; 0.77
**Health system levels (vs central):**								
Provincial	-0.14	-0.34; 0.07					-0.13	-0.32; 0.06
District	-0.52***	-0.89; -0.14	-0.53***	-0.88; -0.18	-0.58***	-0.93; -0.23	-0.74***	-1.09; -0.39
**Participated in community activities** (Yes vs no)			0.11	-0.03; 0.25			0.10	-0.03; 0.23
**Region (vs Northern):**								
Central			-0.24	-0.54; 0.06	-0.29*	-0.58; 0.00	-0.19	-0.48; 0.09
South	-0.22***	-0.37; -0.06	-0.37***	-0.53; -0.21	-0.27***	-0.43; -0.12	-0.22***	-0.37; -0.07
**Age group** (25 and above vs under 25)	-0.40**	-0.76; -0.03			-0.71***	-1.05; -0.36	-0.39*	-0.85; 0.07

Coef. – coefficient, CI – confidence interval

* *P* < 0.1, ** *P* < 0.05, ***P < 0.01.

## DISCUSSION

This study provides empirical data to elucidate the operational readiness capacities of the grassroots health system in Vietnam. Generally, capacities regarding four assessed criteria were at moderate levels, revealing vulnerability to the risk of epidemic events of the grassroots health system. Moreover, our findings indicate that these capacities in the rural areas, in the South, and at the district level were more likely to be vulnerable compared to their counterparts. Our findings provide useful insights for effective strategies to strengthen the capacity of the grassroots health system in the long term. In the short term, practicing precautionary measures to avoid adding the burden on the health system and mobilizing human resources, as well as medical equipment are all needed to fight against COVID-19 in Vietnam.

The scores rated in our survey were relatively parallel with average scores of the capacity for human resources and health service provision of the Vietnamese health system reported in the IHR SPAR 2018 (60%)[18]. Moreover, in the IHR SPAR 2018, data were recorded at the national level while ours were at the grassroots level. This study, therefore, enriches the understanding of the validity of the IHR SPAR 2018 and makes it contextually appropriate not only at the national level but also at the grassroots level in Vietnam.

Previous studies showed that the lower the IHR SPAR scores, the higher the risk of potential health emergencies, including COVID-19 [21,22]. Given the moderate scores of operational readiness capacities, this study also indicates the necessity to strengthen emergency response capacity in the long term. As a lesson learned from prior natural disasters and epidemics, providing additional personnel with adequate competencies, proper knowledge and skills [23], developing training programs in epidemic preparedness and management of mass casualty incidents [24,25], and ensuring an adequate supply of medical equipment for frontlines [26,27] are critical to minimize the risk of health emergencies. In the short term, these findings imply that additional precautionary measures are indispensable for relieving the burden on the grassroots health system. Of note, several simple but effective precautionary measures such as wearing masks, regardless of the presence or absence of symptoms of COVID-19, and washing hands with soap and water frequently have been applied in Vietnam since the early stage of COVID-19.

This study also provides the rationality of prompt and vigorous actions of the Vietnamese Government against COVID-19. Due to the insufficiency of equipment and facilities in addition to limited financial resources, it might not be feasible to implement mass community testing for the SARS-COV-2. In reality, recognizing the vulnerability of the health system, the Vietnamese Government has been responding rapidly to the COVID-19 pandemic since the very first infected case was confirmed on January 22, 2020 [28]. A combination of extensive approaches encompasses immediate quarantining infected people and their direct contacts, lockdown areas nearby their residential places, contact tracing, and keeping indirect connections under surveillance. Besides, temperature screening at arrival gates in the airports, at large buildings, and at shopping malls had been performed. School closures and cancellations of festivals, events, and other gathering activities have also been implemented. To minimize imported cases, Vietnam has also deployed rigorous border control measures, including visa entry halting, mandatory health declaration, and enforcement of 14-day quarantine for all incoming travelers to Vietnam [19,29]. When there were more than 200 confirmed cases, a 14-day social distancing requirement had been applied in the entire nation since April 1, 2020. Within the context of Vietnam, these strategies were considered necessary to support the endurance of the fragile health system.

Upon analysis, the operational readiness capacities of the health system in rural regions, in the South, and at the district level were more likely to be limited compared to their counterparts. The disparities among regions in Vietnam have been relatively similar to findings in China [27] and in Ethiopia [30]. This finding suggests that decision-makers should consider a mechanism for managing limited-resources allocation and mobilization among regions as well as a strategy to engage communities in improving locally responsive health care [31]. At the time of writing this paper, the entire Vietnamese population regardless of their socio-economic status, including soldiers, businessmen, scholars, and students are currently support health care workers in the battle against COVID-19. There are many military buildings, university facilities, and dormitories that have been requisitioned as quarantine camps and many temporary hospitals have been rapidly built to support the insufficiency of health care facilities.

There are several implications drawn from this study. First, given moderate scores of operational readiness capacities, strategies to strengthen the capacity of the grassroots health system such as improve the quality and quantity of health care professionals, administrative and logistics staffs, developing training programs, and ensuring an adequate supply of medical equipment are needed in the long term. Second, in the short term, practicing precautionary measures, planning of rapid coordinated responses with community engagement, and mobilizing human resources and medical equipment to relieve the burden on the grassroots health system are necessary to control COVID-19 in Vietnam successfully.

The strength of this study is conducting on large sample size and coverage from the North to the South of Vietnam. This is considered as a condition to enhance the generalizability of study findings. However, this study also contains several limitations. First, we recruited respondents who were referred online rather than randomly selected from a nationally representative sample frame. In addition, the cross-sectional study design is unable to identify the cause-effect relationship between dependent variables and independent ones. Also, the use of self-administration questionnaires did not allowed researchers to explain unclear terms to respondents. Finally, self-reporting data may cause recall bias.

## CONCLUSIONS

According to empirical data, this study reveals the vulnerability of the grassroots health system in Vietnam and provides the rationality of prompt and vigorous actions of the Vietnamese Government against COVID-19. Findings also offer useful insights for effective strategies to strengthen the grassroots health system in the long term. In the short term, practicing precautionary measures and mobilizing human resources, as well as medical equipment, are necessary for successful COVID-19 containment in Vietnam.

## References

[1] World Health Organization. Coronavirus disease (COVID-19) Pandemic. 2020. Available: https://www.who.int/emergencies/diseases/novel-coronavirus-2019. Accessed: 18 April 2020.

[2] World Health Organization. Q&A on coronaviruses (COVID-19). 2020. Available: https://www.who.int/news-room/q-a-detail/q-a-coronaviruses. Accessed: 18 April 2020.

[3] World Health Organization. Pneumonia of unknown cause – China. 2020. Available: https://www.who.int/csr/don/05-january-2020-pneumonia-of-unkown-cause-china/en/. Accessed: 18 April 2020.

[4] World Health Organization. WHO Timeline - COVID-19. 2020. Available: https://www.who.int/news-room/detail/08-04-2020-who-timeline—covid-19. Accessed: 18 April 2020.

[5] Wang C Horby PW Hayden FG Gao GF A novel coronavirus outbreak of global health concern. Lancet. 2020;395:470-3. . 10.1016/S0140-6736(20)30185-9 31986257 PMC7135038

[6] Ciotti M Angeletti S Minieri M Giovannetti M Benvenuto D Pascarella S COVID-19 outbreak: An overview. Chemotherapy. 2020;1-9. Online before print. 10.1159/000507423 32259829 PMC7179549

[7] Torales J O’Higgins M Castaldelli-Maia JM Ventriglio A The outbreak of COVID-19 coronavirus and its impact on global mental health. Int J Soc Psychiatry. 2020;20764020915212. Online before print. . 10.1177/0020764020915212 32233719

[8] Tan BYQ Chew NWS Lee GKH Jing M Goh Y Yeo LLL Psychological Impact of the COVID-19 Pandemic on Health Care Workers in Singapore. Ann Intern Med. 2020;M20-1083. Online before print. 10.7326/M20-1083 32251513 PMC7143149

[9] World Health Organization. Coronavirus (COVID-19). 2020. Available: https://covid19.who.int/. Accessed: 18 April 2020.

[10] Wang C Pan R Wan X Tan Y Xu L Ho CS Immediate psychological responses and associated factors during the initial stage of the 2019 Coronavirus Disease (COVID-19) Epidemic among the general population in China. Int J Environ Res Public Health. 2020;17:1729. 10.3390/ijerph17051729 32155789 PMC7084952

[11] Wang C Pan R Wan X Tan Y Xu L McIntyre RS A longitudinal study on the mental health of general population during the COVID-19 Epidemic in China. Brain Behav Immun. 2020. Epub ahead of print. 10.1016/j.bbi.2020.04.028 32298802 PMC7153528

[12] Nicolás ES. Coronavirus: EU ministers urge members to share supplies. 2020. Available: https://euobserver.com/social/147659. Accessed: 18 April 2020.

[13] Anderson M McKee M Mossialos E Covid-19 exposes weaknesses in European response to outbreaks. BMJ. 2020;368:m1075. 10.1136/bmj.m1075 32188590

[14] Scott D. Coronavirus is exposing all of the weaknesses in the US health system. 2020. Available: https://www.vox.com/policy-and-politics/2020/3/16/21173766/coronavirus-covid-19-us-cases-health-care-system. Accessed: 14 April 2020.

[15] Emanuel EJ Persad G Upshur R Thome B Parker M Glickman A Fair allocation of scarce medical resources in the time of Covid-19. N Engl J Med. 2020. Epub ahead of print. 10.1056/NEJMsb2005114 32202722

[16] Paterlini M On the front lines of coronavirus: the Italian response to covid-19. BMJ. 2020;368:m1065. 10.1136/bmj.m1065 32179517

[17] Ranney ML Griffeth V Jha AK Critical supply shortages - The need for ventilators and Personal Protective Equipment during the Covid-19 Pandemic. N Engl J Med. 2020;382:e41. 10.1056/NEJMp2006141 32212516

[18] World Health Organization. IHR States Parties Self-Assessment Annual Reporting (SPAR). 2020. Available: https://extranet.who.int/sph/spar/spar/360. Accessed: 18 April 2020.

[19] Nguyen THD Vu DC Summary of the COVID-19 outbreak in Vietnam - Lessons and suggestions. Travel Med Infect Dis. 2020;101651. Online before print. 10.1016/j.tmaid.2020.101651 32247928 PMC7146658

[20] Statistics on COVID-19 disease situation Vietnam. 2020. Available: https://ncov.moh.gov.vn/. Accessed: 18 April 2020.

[21] Kandel N Chungong S Omaar A Xing J Health security capacities in the context of COVID-19 outbreak: an analysis of International Health Regulations annual report data from 182 countries. Lancet. 2020;395:1047-53. 10.1016/S0140-6736(20)30553-5 32199075 PMC7271261

[22] Gilbert M Pullano G Pinotti F Valdano E Poletto C Boëlle PY Preparedness and vulnerability of African countries against importations of COVID-19: a modelling study. Lancet. 2020;395:871-7. 10.1016/S0140-6736(20)30411-6 32087820 PMC7159277

[23] Van Minh H Tuan Anh T Rocklöv J Bao Giang K Trang le Q Sahlen KG Primary healthcare system capacities for responding to storm and flood-related health problems: a case study from a rural district in central Vietnam. Glob Health Action. 2014;7:23007. 10.3402/gha.v7.23007 25511879 PMC4265642

[24] Bemah P Baller A Cooper C Massaquoi M Skrip L Rude JM Strengthening healthcare workforce capacity during and post Ebola outbreaks in Liberia: an innovative and effective approach to epidemic preparedness and response. Pan Afr Med J. 2019;33 Suppl 2:9. 10.11604/pamj.supp.2019.33.2.17619 31402967 PMC6675930

[25] Oji MO Haile M Baller A Tremblay N Mahmoud N Gasasira A Implementing infection prevention and control capacity building strategies within the context of Ebola outbreak in a “Hard-to-Reach” area of Liberia. Pan Afr Med J. 2018;31:107. 10.11604/pamj.2018.31.107.15517 31037168 PMC6462381

[26] Maves RC Jamros CM Smith AG Intensive Care Unit Preparedness During Pandemics and Other Biological Threats. Crit Care Clin. 2019;35:609-18. 10.1016/j.ccc.2019.06.001 31445608 PMC7134984

[27] Tong MX Hansen A Hanson-Easey S Xiang J Cameron S Liu Q China’s capacity of hospitals to deal with infectious diseases in the context of climate change. Soc Sci Med. 2018;206:60-6. 10.1016/j.socscimed.2018.04.021 29684649 PMC7116943

[28] Phan LT Nguyen TV Luong QC Nguyen TV Nguyen HT Le HQ Importation and Human-to-Human Transmission of a Novel Coronavirus in Vietnam. N Engl J Med. 2020;382:872-4. 10.1056/NEJMc2001272 31991079 PMC7121428

[29] Dinh L Dinh P Nguyen PDM Nguyen DHN Hoang T Vietnam's response to COVID-19: Prompt and proactive actions. J Travel Med. 2020;taaa047. 10.1093/jtm/taaa047 32297929 PMC7184391

[30] Deribew A Biadgilign S Berhanu D Defar A Deribe K Tekle E Capacity of health facilities for diagnosis and treatment of HIV/AIDS in Ethiopia. BMC Health Serv Res. 2018;18:535. 10.1186/s12913-018-3347-8 29996821 PMC6042210

[31] Kenny A Hyett N Sawtell J Dickson-Swift V Farmer J O’Meara P Community participation in rural health: a scoping review. BMC Health Serv Res. 2013;13:64. 10.1186/1472-6963-13-64 23414561 PMC3583801

